# Selected Population-based Oral Health Assessment Following Usage of E-Cigarettes and Combustible Cigarettes

**DOI:** 10.3290/j.ohpd.c_2292

**Published:** 2025-11-12

**Authors:** Xiao Li, Jilong Liu, Dong Yang, Duo Wang, Qiulin Yue, Song Zhang, Jing Xu, Xingtao Jiang, Qun Su, Lei Sun, Baojun Li, Kunlun Li, Le Su, Lin Zhao

**Affiliations:** a Xiao Li Postgraduate Student, State Key Laboratory of Biobased Material and Green Papermaking, School of Bioengineering, Qilu University of Technology, Shandong Academy of Sciences, Jinan, China. State Key Laboratory of Biobased Material and Green Papermaking, School of Bioengineering, Qilu University of Technology, Shandong Academy of Sciences, Jinan, China. Experimental design, sample collection, main experimental work, drafted the manuscript.; b Jilong Liu Postgraduate Student,State Key Laboratory of Biobased Material and Green Papermaking, School of Bioengineering, Qilu University of Technology, Shandong Academy of Sciences, Jinan, China. Assisted with the experiments.; c Dong Jang Postgraduate Student, State Key Laboratory of Biobased Material and Green Papermaking, School of Bioengineering, Qilu University of Technology, Shandong Academy of Sciences, Jinan, China. Data processing and analysis.; d Duo Wang Postgraduate Student, State Key Laboratory of Biobased Material and Green Papermaking, School of Bioengineering, Qilu University of Technology, Shandong Academy of Sciences, Jinan, China. Data processing and analysis.; e Qiulin Yue Lecturer, State Key Laboratory of Biobased Material and Green Papermaking, School of Bioengineering, Qilu University of Technology, Shandong Academy of Sciences, Jinan, China. Data interpretation.; f Song Zhang Associate Professor, State Key Laboratory of Biobased Material and Green Papermaking, School of Bioengineering, Qilu University of Technology, Shandong Academy of Sciences, Jinan, China. Data interpretation.; g Jing Xu Senior Engineer, Shenzhen RELX Tech Co, Ltd, Shenzhen, China. Provided financial support.; h Xingtao Jiang Senior Engineer, Shenzhen RELX Tech Co, Ltd, Shenzhen, China. Provided financial support.; i Qun Su Associate Professor, Shandong Baoyuan Biotechnology Co, Ltd, Yantai, PR China. Provided financial support.; j Lei Sun Senior Engineer, Shandong Baoyuan Biotechnology Co, Ltd, Yantai, PR China. Provided financial support.; k Baojun Li Senior Engineer, Shandong Danhe Biotechnology Co, Ltd, Jinan, PR China. Provided financial support.; l Kunlun Li Senior Engineer, Shengshengxiangrong (Shandong) Biotechnology Co., Ltd., Jinan, PR China. Provided financial support.; m Le Su Senior Engineer, State Key Laboratory of Biobased Material and Green Papermaking, School of Bioengineering, Qilu University of Technology, Shandong Academy of Sciences, Jinan, China. Critically revised the manuscript and approved final version to be published.; n Lin Zhao Professor, State Key Laboratory of Biobased Material and Green Papermaking, School of Bioengineering, Qilu University of Technology, Shandong Academy of Sciences, Jinan, China. Critically revised the manuscript and approved final version to be published. All authors contributed substantially to concept and design.

**Keywords:** electronic cigarettes, oral health, population analysis, traditional cigarettes.

## Abstract

**Purpose:**

To compare oral health following e-cigarette and cigarette use in a Chinese male population.

**Materials and Methods:**

In this study, we selected 83 adult Chinese males aged between 18-35 years of age, including 31 regular traditional cigarette smokers, 20 regular e-cigarette users, and 32 never-smokers and never-vapers. Their clinical periodontal indicators (bleeding on probing, probing depth, clinical attachment loss) and salivary levels of interleukin (IL)-6, IL-8, IL-1β and cotinine were measured and compared.

**Results:**

In this selected population, traditional cigarette smokers (CS) had statistically significantly higher bleeding on probing and clinical attachment loss than did ES and NS. The probing depths and IL-6, IL-8, and IL-1β levels were statistically significantly higher in CS than in e-cigarette users (ES) and never-smokers (NS).

**Conclusions:**

This study demonstrated that the oral health status of CS was worse than that of NS and ES in adult males aged 18–35 years.

In recent years, health awareness in the general population has been increasing, and the harm of cigarette smoking is recognized by more people. Tobacco product use declined by about 0.6 billion in 2018 compared to in 2000.^28^ Cigarette smoke contains a variety of chemicals, including some toxic substances and carcinogens,^1^ and is an important factor affecting systemic health, damaging almost every organ in the body. Smoking increases the risks of cancer and cardiovascular diseases (e.g., atherosclerosis), and also affects oral health.^1,2,22,40^ To reduce the harm of cigarette smoking, some smokers are looking for alternatives: electronic cigarettes (e-cigarettes) and heat-not-burn tobacco products have become available. The sales of e-cigarettes had increased rapidly since their launch almost a decade ago.^18^ A typical e-cigarette is a battery-powered nicotine delivery system that does not contain tobacco and consists of a heater, an e-liquid cartridge with different ingredients, and an atomizer. The heating atomizer evaporates the e-liquid in the cartridge into an aerosol that can be inhaled.^18^ Unlike combustible cigarettes that burn tobacco, e-cigarettes generate an aerosol to simulate a smoking-like action and feeling of nicotine. E-cigarette aerosols do not contain tobacco tar as compared to cigarette smoke. The aerosols of e-cigarettes have been found to contain some toxicants and carcinogens, such as nickel, lead, and other heavy metals, which accumulate in the body. Polycyclic aromatic hydrocarbons and aldehydes have strong carcinogenicity. Volatile organic compounds such as benzene are also toxic. However, the amounts and types are below that of cigarette smoke.^17,39^

Oral health is very important in our daily life. It is not only related to the quality of life but also has an important relationship to overall health.^11,32,33,46^ Periodontal diseases are common oral diseases affecting up to 90% of the world’s population. It usually starts with gingivitis and progresses to periodontitis, which eventually leads to tooth loss.^6,36,47^ There are many factors affecting periodontal disease, including bacterial biofilm, environmental exposure, genetics, and smoking; the latter is a preventable risk factor. Studies have shown that smokers are more likely to suffer from periodontal disease than never-smokers, and smoking can increase the risk of developing periodontal disease by 85%.^27^ Smoking also causes an imbalance in the oral microbiome, leading to the development of periodontal disease.^3^

Research results on the safety of e-cigarettes have been controversial. Short-term in-vitro studies using cell models have shown that e-cigarette exposures are harmful to oral cells, but less so than cigarette smoking. E-cigarette aerosol could change the morphology of gingival epithelial cells and induce cell apoptosis. Studies comparing e-cigarette and traditional cigarette use showed that e-cigarette aerosol induced less damage to gingival fibroblasts and gingival mesenchymal stem cells than did traditional cigarette smoke.^9,37,45^ So far, clinical studies on the oral health effects of e-cigarette and traditional cigarette usages are few and have limitations.

Given the importance of oral health, clinical studies on the long-term usage effects of e-cigarettes and traditional cigarettes on human oral cells and on oral health are needed. Thus, the purpose of this study was to compare the effects of e-cigarette use vs cigarette smoking on the periodontium in a sub-population of adult Chinese males between the ages of 18–35 years.

## MATERIALS AND METHODS

### Ethical Approval 

This study was approved by the Institutional Review Board of Shandong Danhe Biotechnology (DH20230116; Jinan, China). All participants were informed in writing before the study began that the study was voluntary and subjects could withdraw from the study at any time. All the participants read and signed the informed consent.

### Participants and Inclusion/Exclusion Criteria

The participants in the study included 32 never-smokers, 31 traditional cigarette smokers and 20 e-cigarette users, all of whom were all adult Chinese males between the ages of 18 and 35.

1. Never-smokers (NS): never used tobacco products in any form prior to the study.
2. Traditional cigarette smokers (CS): smoked only traditional cigarettes for at least 12 months prior to the study. Their cigarette smoking status was recorded in a questionnaire but not independently checked.
3. E-cigarette users (ES): only used e-cigarettes for 12 months prior to the study. Their vaping status was recoded in a questionnaire but not independently checked.

Exclusion criteria included any of the following conditions that existed before or during the experiment: (1) chronic dry mouth; (2) periodontal pocket ≥ 4 mm; (3) untreated caries lesions or oral abscesses; (4) precancerous or cancerous lesions; (5) Candidiasis; (6) bad breath; (7) more than 8 teeth missing due to various reasons; (8) bleeding at more than 10% of sites probed.

### Questionnaire

A questionnaire was prepared for all participants to record their age, ethnicity, gender, daily brushing frequency, smoking frequency, duration and daily frequency of smoking and vaping, and family history of smoking.

### Collecting Saliva and Calculating Saliva Flow Rate 

Participants were asked to open their mouths slightly and let saliva flow into a saliva trap for 5 min. Saliva was collected between 8:30 AM and 9:00 AM, with subjects having fasted since awakening. The salivary flow rate (SFR) was calculated by dividing the total volume of saliva collected by time and was expressed in ml/min.^29^ Part of the collected saliva was used to measure inflammatory markers, and the rest was frozen at -80°C.

### Determination of Inflammatory Markers and Cotinine Content

Part of the saliva was centrifuged at 1000 rpm at 4°C for 20 min to obtain the supernatant. The contents of IL-6, IL-8, IL-1β and cotinine were determined according to the instructions of the kits (1110602, 1110802, 579409, Dakewei Biotechnology; Beijing, China; MB-16068A, Meibiao Biotechnology; Jiangsu, China).

### Determination of Periodontal Indicators

In all participants, BOP, PD, PI, and CAL were measured.^4,12,13, 19,30^ A periodontal probe (L3-098, WAHA; Okinawa, Japan) was used to measure BOP, PD and CAL of each tooth at six sites in the distal, central and mesial buccal (lip) and lingual sides. A total of 8 teeth were tested per subject.

Determination of BOP: the tip of the periodontal probe was placed 1 mm below the gingival margin, and slid gently along the gingival margin with about 20–25 grams of force. Bleeding was observed and recorded.

PD measurement: A periodontal probe was used to measure the depth from the bottom of the periodontal pocket to the gingival margin with a force of about 25 grams, and PD was read as the graduation mark on the probe.

Determination of CAL: After measuring the depth of the periodontal pocket, the location of the cementum boundary was explored when the tip of the probe was withdrawn along the root surface. The distance from the cementum boundary to the gingival margin was measured. The degree of attachment loss was calculated by subtracting the distance from the depth of the pocket. If the two numbers were reduced to zero, or the cementum boundary could not be reached, then no attachment was lost.

A cotton swab was dipped into a plaque indicator (Huayou Medical Instruments; Guangzhou, China) and gently smeared on the surface of 8 teeth. Gargling was performed immediately after the smear to reveal dental plaque. The plaque score for each tooth was recorded, and the score was added and divided by the number of teeth to give the PI for each participant.

PI was scored using a five-point method, as follows:

0 = No plaque
1 = Dotted around the neck edge of the tooth
2 = A thin continuous band of plaque at the neck edge of the tooth, not exceeding 1 mm in width
3 = A wide continuous plaque band on the neck of the tooth, more than 1 mm in width but less than 1/3 of the entire tooth surface
4 = Dental plaque covering at least 1/3 of the entire dental surface, less than 2/3 of the dental surface
5 = Plaque covering 2/3 or more of the entire dental surface.

### Data Analysis

Data analysis was performed using Prism (Version 9, Graphd Prism; San Diego, CA, USA). One-way ANOVA was used to compare the periodontal indices and the contents of cotinine, IL-6, IL-8 and IL-1β among the NS, CS, and ES groups. For multiple comparisons, Bonferroni adjusted post-hoc tests were performed. Statistical significance was set at p < 0.05.

## RESULTS

The data used and/or analyzed during the current study are available from the corresponding author on reasonable request.

### Participant Information

Atotal of 83 adult males met the selection criteria and were enrolled. Based on their answers to the questionnaire, they classified into the NS group (32 subjects), CS group (31 subjects), and ES group (20 subjects). The mean age of the NS group was 20.94 ± 2.08 years, that of the CS group was 20.71 ± 1.00 years, and mean age in the ES group was 26.45 ± 3.72 years. No spouses of participants in the three groups smoked. People in the CS group smoked 7 cigarettes a day, and those in the ES group vaped one cigarette cartridge every three days. The traditional cigarettes and e-cigarettes used by the participants were all provided by the authors. The brand of traditional cigarettes is “Hongtashan” and the brand of e-cigarettes is “RELX”, both of which have a nicotine content of 3%. The content of each e-cigarette cartridge is 2 grams, comprising nicotine, benzoic acid, menthol, propylene glycol, propylene glycol, essence, etc. 65.52%, 73.68%, and 76% of the NS, CS, and ES groups had a family history of smoking, respectively (Table 1).

**Table 1:** Characteristics and periodontal indicators of the study population in each group

Parameters	NS	CS	ES
Number	32	31	20
Gender (male)	32	31	20
Age in years (mean ± SD)	20.94 ± 2.08	20.71 ± 1.00	26.45 ± 3.72
Mean smoking	–	7 cigarettes a day	One cartridge every three days
BOP	Yes	No	Yes	No	Yes	No
15%	85%	43.48%	56.52%	18.52%	81.48%
CAL	Yes	No	Yes	No	Yes	No
7.5%	92.5%	43.48%	56.52%	11.11%	w
Family history of smoking	65.52%	73.68%	76%


### Determination of Clinical Periodontal Indicators

The measurement of BOP, CAL, PD and PI in each group showed that 15% of the subjects in the NS group had gingival bleeding and 7.5% had attachment losses. For the CS group, gingival bleeding and attachment losses accounted for 43.48% and 43.48% of the total CS group, respectively. In the ES group, 18.52% had gingival bleeding and 11.11% had attachment loss (Table 1).

**Fig 1:**
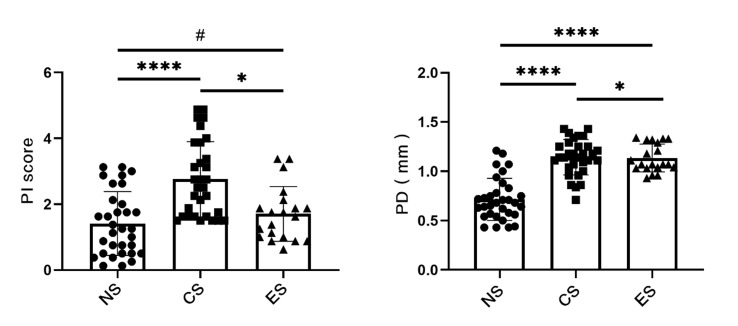
Mean and individual values of PI score and PD. (a) Comparison of PI scores in NS group, CS group and ES group. (b) Comparison of PD in NS group, CS group and ES group. NS: never-smokers. CS: traditional cigarette smokers. ES: e-cigarette smokers. PI: plaque index. PD: probing depth. (#p > 0.05, *p < 0.05, ****p < 0.0001).

The PI scores of the NS group ranged from 0.125 to 3.125 (mean = 1.41); the CS group had a PI score ranging from 1.5 to 4.875 (mean = 2.76), and the PI score of the ES group was between 0.625 and 3.375 (mean = 1.71). The CS group had statistically significantly higher PI scores than those in the NS (p < 0.0001) and ES groups (p< 0.05), while there was no statistically significant difference between the PI scores in the ES and NS groups (Fig 1a). For PD, the results in the NS group ranged from 0.43 mm to 1.21mm (mean = 0.72 mm). PD in the CS group ranged from 0.71–1.43 mm (mean = 1.14 mm); in the ES group, it ranged from 0.93–1.34 mm (mean = 1.135 mm). The CS group had statistically significantly higher PDs than those of the NS (p < 0.0001) and ES groups (p < 0.05), and the ES group had statistically significantly higher PD than the NS group (p < 0.0001) (Fig 1b).

### Measurement of Salivary Flow Rate (SFR)

**Fig 2:**
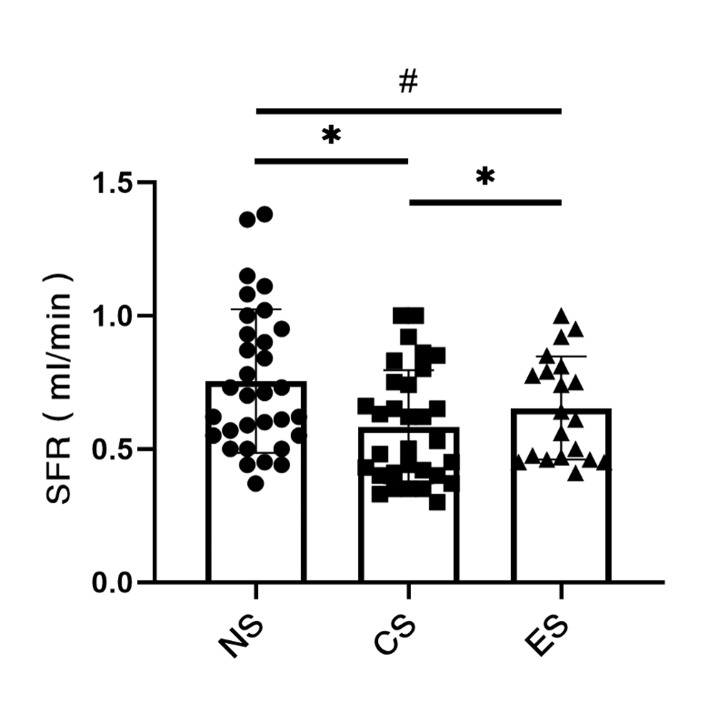
The mean and individual values of SFR in each group. NS: never-smokers. CS: traditional cigarette smokers. ES: e-cigarette smokers. SFR: salivary flow rate. (#p > 0.05, *p < 0.05).

SFRs were found to be between 0.37 and 1.38 ml/min in the NS group, between 0.30 and 1.00 ml/min in the CS group, and between 0.41 and 1.00 ml/min in the EC group. The mean SFR was 0.74 ml/min in the NS group, 0.59 ml/min in the CS group, and 0.67 ml/min in the ES group. As can be seen from Fig 2, compared with the NS group, the SFR of the CS group decreased statistically significantly (p < 0.05). There were no statistically significant changes in the ES group compared to the NS group.

### Examination of Inflammatory Cytokines and Cotinine Levels 

**Fig 3:**
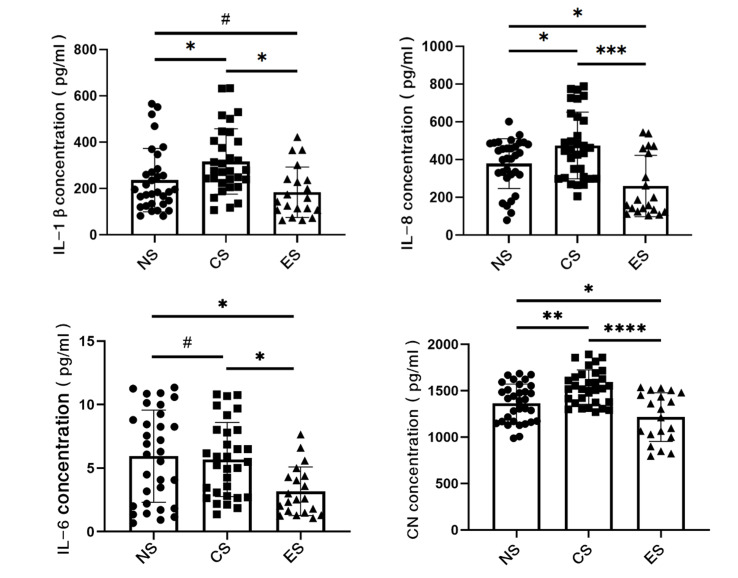
The mean and individual values of inflammatory factors and cotinine in each group. (a) Comparison of IL-1β content in NS group, CS group and ES group. (b) Comparison of IL-8 content in NS group, CS group and ES group. (c) Comparison of IL-6 content in NS group, CS group and ES group. (d) Comparison of cotinine content in NS group, CS group and ES group. NS: never-smokers. CS: traditional cigarette smokers. ES: e-cigarette smokers. CN: cotinine. (#p > 0.05, *p < 0.05, **p < 0.01, ***p < 0.001, ****p < 0.0001).

ELISA was used to measure inflammatory markers and cotinine levels in saliva. For IL-1β level, the average content of IL-1β in NS group was 236.48 pg/ml, in the CS group it was 317.25 pg/ml, and in the ES group 184.16 pg/ml. The content of IL-1β in the CS group was statistically significantly higher than that in the NS and ES groups (p < 0.05). There was no statistically significant change in the content of IL-1β in ES group compared with the NS group (Fig 3a). As for the level of IL-8, the average content in the NS group was 379.27 pg/ml, CS group was 475.13 pg/ml, and ES group was 260.17 pg/ml. The content of IL-8 in CS group was statistically significantly higher than that in the NS (p < 0.05) and ES groupx (p < 0.001), while the content of IL-8 in ES group was statistically significantly lower than that in NS group (p < 0.05) (Fig 3b). Regarding IL-6, the average content in the NS group was 5.95 pg/ml, 5.68 pg/ml in the CS group, and 3.18 pg/ml in the ES group. There was no statistically significant difference between the CS and NS groups, but the content of IL-6 in the ES group was statistically significantly lower than that of the CS and NS groups (p < 0.05) (Fig 3c). For the content of cotinine, the average content of cotinine in NS group was 1364.50 pg/ml, that in CS group was 1539.04 pg/ml, and that in ES group was 1215.54 pg/ml. The content of the CS group was statistically significantly higher than that of NS (p < 0.01) and ES groups (p < 0.0001), and the content of ES group was statistically significantly lower than that of NS group (p < 0.05) (Fig 3d).

## DISCUSSION

With e-cigarettes’ increasing popularity, more studies are needed to evaluate their effects on general and oral health.^1^ Compared to traditional cigarettes, e-cigarettes do not contain tobacco tar and have fewer toxicants and other chemical ingredients, but the aerosol produced by e-cigarettes still contains some toxic compounds, such as carbonyls and heavy metals.^10^ Some in-vitro studies have shown that e-cigarette aerosols cause damage to oral cells, but the damage is less than that from traditional cigarettes.^5,37,45^ However, in a clinical setting, a Korean study pointed out that both e-cigarettes and traditional cigarettes are closely related to the occurrence and development of periodontal disease.^20^ Another study showed that periodontal inflammation was more severe in people who smoked traditional cigarettes than in people who used e-cigarettes.^21^

Young adults ages 18–30 use e-cigarettes more than tobacco products.^8^ In our study, we measured the periodontal parameters and the levels of inflammatory factors and cotinine in saliva of 32 NS, 31 CS and 20 ES subjects, and compared the effects of e-cigarette and traditional cigarette on periodontal and oral health for adult males aged between 20 and 30 years.

Clinical and radiological methods are commonly used to assess the severity of periodontitis.^29^ In persons suffering from periodontal diseases, pathogenic microorganisms in the mouth cause inflammation and the formation of dental plaque.^36^ The accumulation of pathogenic microorganisms will form periodontal pockets, which will lead to the loss of gum tissue attachment.^36^ Gingival hemorrhage (BOP) is an inflammation of the connective tissue of the gums.^32^ Studies have found that traditional cigarettes and e-cigarettes statistically significantly reduced BOP and that traditional cigarettes statistically significantly increased PD, PI, and CAL, while e-cigarettes caused no statistically significant changes in these indicators.^19,29^ The results of the present study showed a statistically significantly higher number of people with BOP and CAL in the CS group than in the NS and ES groups, while the number of people with BOP and CAL in the ES group very similar to that in the NS group. The PI score and PD in the CS group were statistically significantly higher than in the ES and NS groups. As for the results of PI scores, we selected participants with similar oral care through questionnaires in the early stage. In order to exclude other factors, participants were explicitly told to brush their teeth once every morning and evening, and record their smoking times and inhalation time before the experiment began. The high PI value of CS group is considered to be due to the fact that substances such as tar are produced when traditional cigarettes are burned, and these substances adhere to the tooth surface. In addition, studies have shown that smokers have an increased tendency to form adhesive biofilms and altered salivary pH, which is conducive to the proliferation of harmful bacteria and is more favorable for the formation of dental plaque.^14,44^ The oral health status in this study gradually deteriorated with age and smoking duration, presumably due to the younger age and fewer years of smoking of our selected population.^29^ These data suggested that traditional cigarettes are more damaging to periodontal status.

Saliva is essential for maintaining oral health.^15^ Studies have found that individuals with low saliva flow have more plaque, and plaque buildup accelerates the development of gum inflammation.^41^ Reduced saliva production also increases the risk of dental caries.^42^ The present results showed that traditional cigarettes statistically significantly reduced the salivary flow rate, while there was no statistically significant change in the e-cigarette group. Our findings on saliva flow rates are consistent with those of Meghani et al.^31^ We hypothesized that the reduced salivary secretion in the CS group was due to smoking. This indicates that the oral condition of CS was worse than ES, and the risk of caries was greater in CS.

During the development of oral inflammation, macrophages trigger gingival fibroblasts by releasing cytokines (e.g., IL-1β, TNF-α), thereby increasing the release of IL-6 and IL-11 cytokines; excessive inflammatory factors can exacerbate periodontal tissue damage.^25^ In our results, the levels of inflammatory factors were statistically significantly higher in the CS group than in the NS and ES groups, and the levels of IL-8 and IL-6 were statistically significantly lower in the ES group than in the NS group. In a study by Sameer et al, smoking traditional cigarettes statistically significantly increased IL-6 and IL-8 levels, while the use of e-cigarettes did not.^29^ The ingredients in traditional cigarettes are different from those of e-cigarettes: cigarette smoke contains mainly tar, nicotine, carbon monoxide and other substances, whereas the vapor of e-cigarettes contains glycerin, propylene glycol, flavoring agents, etc.^19,38^ We speculated that the vape fluid of the e-cigarette selected for this experiment may contain certain components that affect the content of inflammatory factors. Studies have shown that menthol has anti-inflammatory effects. Thus, we speculated that menthol components in tobacco oil reduced the content of inflammatory factors in ES group.^7^ In addition, when asked at the time of the experiment, some participants in the NS group reported having mouth ulcers, which may have contributed to the higher inflammatory factor in the NS group.

Cotinine is the main metabolite of nicotine (70%-80%), and nicotine metabolism takes place in the liver.^34^ Aside from the effects of nicotine on heart rate, studies have found little evidence that the development of smoking-related diseases is due to nicotine exposure.^23^ In contrast, many studies have shown that cotinine can lead to the occurrence of respiratory diseases, such as asthma;^24^ this suggests that more attention should be paid to levels of the nicotine metabolite cotinine.^23^ Compared with the levels of salivary cotinine reported in the literature (traditional smokers, 370.29 ± 308.08 ng/ml; e-cigarette users, 349.56 ± 239.04 ng/ml),^16^ the average content of cotinine in CS group and ES group in our study was not high (CS, 1539.04 pg/ml; ES, 1215.54 pg/ml). This could be due to most of the particpants in the study by Hasan et al^16^ being around 34 years old, who had smoked for at least a year. Most of our volunteers were around 20 years old and had smoked for a shorter period of time, which would explain the lower levels of cotinine. Additionally, cotinine content in the CS group was statistically significantly higher than that in the NS and ES groups, while it was lower in the ES group. Both the cigarettes and e-cigarettes we provided to our participants contained 3% nicotine, and studies have shown that long-term e-cigarette users may obtain roughly similar levels of nicotine in their saliva compared to smokers who only use combustible cigarettes.^43^ We speculated that certain substances may affect the metabolism of nicotine. It is suggested that some of the compounds contained in grapefruit juice and menthol could inhibit nicotine metabolism by directly interacting with CYP2A6 enzyme.^34^ Menthol is also a popular flavor additive for cigarettes.^25^ We speculate that menthol in the e-cigarette liquid used by the study subjects may affect the metabolism of nicotine, resulting in the normal conversion of nicotine to cotinine, resulting in lower cotinine levels in the ES group. Interestingly, cotinine was also present in the saliva of the NS group. Cotinine levels in saliva from passive smoking populations in a previous study were similar to that in the NS group we examined.^35^ It has been reported that the level of cotinine in saliva of passive smokers is about 1000 pg/ml, which is similar to the results in the NS group. Prior to commencement of the present study, the family smoking histories of all volunteers were documented, showing that family smoking history was similar in all three groups. This suggests that the cotinine content in NS group might be due to familial exposure to second-hand smoke.

This study has some limitations. Bearing in mind that the severity of periodontal disease is proportional to the age of smoking and the duration of smoking, the population selected for this study had been smoking for a relatively short period of time. Only light smokers were included; heavy smokers were not recruited. In addition, the selected indicators were measured at just one time point. To track the changes longitudinally, these indicators should also be tested at multiple time points.

## CONCLUSION

In this study, traditional cigarette users had statistically significantly higher BOP, CAL, PD, SFR and PI scores than e-cigarette users and never-smokers/never-vapers. The BOP, CAL, PI scores of e-cigarette users and never-smokers did not change statistically significantly, and the SFR and PD of e-cigarette users were statistically significantly higher than that of never-smokers. Traditional cigarette users had statistically significantly higher levels of inflammatory cytokines and cotinine than never-smokers and e-cigarette users, while e-cigarette users had statistically significantly lower levels of IL-8, IL-6 and cotinine than traditional cigarette smokers. Overall, in our study, e-cigarettes had less impact on oral health than did traditional cigarettes. Our study was more comprehensive than those published so far, involving both the measurement of periodontal indicators in the population and the measurement of inflammatory factors in saliva.

## ACKNOWLEDGEMENTS

The authors declare that they have no competing interests. This work was supported by Yantai Development Zone Science and Technology Leading Talents Project (Grant number 2020CXRC4); Shandong Taishan Leading Talent Project (Grant number LJNY202015); Technology-based small and medium enterprises innovation capacity enhancement project (Grant number 2023TSGC0169); Central leadership of local science and technology development special funds (Grant number YDZX2022181); Qilu University of Science, education and industry integration innovation pilot project (Grant number 2023RCKY210).
